# Empowerment and Employee Well-Being: A Mediation Analysis Study

**DOI:** 10.3390/ijerph18115822

**Published:** 2021-05-28

**Authors:** Juan A. Marin-Garcia, Tomas Bonavia

**Affiliations:** 1 ROGLE-Department Organización de Empresas, Universitat Politècnica de València, 46022 Valencia, Spain; 2 Department de Psicología Social, Facultat de Psicologia, Universitat de València, 46010 Valencia, Spain; tomas.bonavia@uv.es

**Keywords:** empowering leadership, empowerment at work, psychological health, physical health, Europe

## Abstract

This study examines the relationship between structural and psychological empowerment and its effects on employees’ psychological, physical, and social well-being. Despite the quantity of previously published works, empirical evidence about these relationships in the workplace is scarce. We developed a mediation model in which structural empowerment predicts employee well-being via psychological empowerment. We based our study on the EU-27 data from the 6th European Working Conditions Survey (EWCS). Data were collected from a questionnaire administered face-to-face to a random sample of employees and the self-employed representative of the working population in the European Union (number of valid responses in this study: 23,468). The effects of the relationships among the variables considered were evaluated using Partial Least Squares (PLS). Results indicate that structural empowerment was positively related to psychological empowerment, which was positively related to job satisfaction, work engagement, and social well-being. The expected relationships for work stress and physical well-being were not found.

## 1. Introduction

The papers of the Special Issue “High-Performance Work Practices and Kaizen: How Sustainable Are They?” analyze the relationship between workplace interventions and their effects on organizations, the environment, or workers’ well-being. Continuing an ongoing academic trend [1,2,3,4,5], a body of work has focused on kaizen or improvement practices. Some of these are centered on healthcare settings [6,7,8,9,10,11], another article analyzes the relationship between kaizen and environmental management, the implementation of cleaner production and green practices [12], and one more analyzes the effects on sustainable product development [13].

On the other hand, in line with the previous investigations [14,15,16,17,18,19,20], the role of diverse High-Performance Work Practices (HPWP) is studied in another body of work, for example, job design [21] or talent management [22] and their connection with the enhancement of social responsibility or the environmental performance of local governments [23,24], work–life balance [25], or employee well-being [26].

In short, HPWP are related to kaizen and worker participation. However, the conditions under which this interaction produces positive effects or can be considered sustainable are unclear [4,17,18,19,27] when considering its long-term contribution to workers’ well-being [20,28], job engagement [29], or organizational commitment [30]. In this empirical research paper, which we will proceed to describe below, we aim to analyze in more detail the relationships that empowerment, which on occasions has been considered an HPWP, has on employee well-being. 

Different studies have linked empowerment with HPWP and kaizen. For example, Maarof and Mahmud [31] highlight employee empowerment as one factor contributing to the successful implementation of kaizen among small and medium-sized enterprises. This is because, by increasing the level of empowerment, more workers will be actively involved in the problem-solving process, thereby increasing their sense of responsibility. In addition to this, Janjić et al. [32] show that employees’ empowerment and development are essential benefits of kaizen implementation. Butler et al. [33] found that the inclusion of employee empowerment in the company’s strategic plan and the development of an employee empowerment culture are critical management decisions that enable sustained continuous improvement of operational performance.

The publications which relate HPWP with empowerment are even more numerous, although Gibson et al. in 2007 highlighted that “research in the domains of empowerment and high-performance/high-involvement practices rarely acknowledge one another” [34] (p. 1468). Nonetheless, despite the differences between both, they share a common interest in showing that practices that increase employee involvement or empowerment should benefit organizations, from the increase of productivity to the improvement of sustainability [35].

Since 2007, the relationships between HPWP and empowerment have not cleared up as much as would have been expected, and a certain degree of confusion persists. Different names are used to refer to HPWP, such as high-involvement work practices or systems or high-commitment work practices [36]. Above all, it is the way of conceptualizing empowerment within HPWP which leads to the greatest confusion. Numerous studies consider empowerment as another element of HPWP. However, in recent years, we have seen a trend that considers empowerment as one of the mediating variables that explains the results obtained when HPWP are implemented. 

Starting from the beginning, the use of empowerment as one of the characteristic practices of HPWP is highlighted, for example, in the different articles published by Osman Karatepe [37]. In these studies, empowerment is considered alongside training and rewards to be among the most important HPWP, empirically linking it with different organizational results. Vatankhah et al. [38], in line with previous studies, point out that empowerment, reward, and promotion are significant indicators of HPWP. These variables act as signals of support that the organization sends to its employees. Atapattu [39] includes, within HPWP, the following: employee empowerment, teamwork, reward system, learning and development, and performance management. All these studies show that empowerment has positive effects on many distinct organizational variables.

However, there is another line of investigation that explains the positive organizational performance outcomes obtained after implementing HPWP and turns to different mediating variables, among which empowerment is found in its different forms. In the article by Liao et al. [40], psychological empowerment fully mediated the relationship between HPWP and knowledge-intensive service performance. Meanwhile, Messersmith et al. [41] showed that the effectiveness of HPWP is partially due to the effect of employee empowerment among other attitudinal variables, confirming empowerment as a critical element of the black box linking HPWP to performance. 

The study of Huertas-Valdivia et al. [42] found that both empowering leadership and psychological empowerment were shown to be independent mediators of the HPWP and engagement relationship. This is to say that their results indicated that the employees who perceive the company as implementing HPWP also report that their managers display more empowering leadership behavior and, in turn, seem to experience a higher level of psychological empowerment, all of which leads to them showing higher levels of work engagement. The study carried out by Arefin et al. [43] also revealed that psychological empowerment mediates the influence of HPWP on job engagement.

In the healthcare sector, various investigations have dealt with the analysis of the relationships between HPWP and empowerment. Bonias et al. [44] discovered that all the components of psychological empowerment, except one, fully mediated the relationship between HPWP and the perception of care quality. In the study by Bartram et al. [45], psychological empowerment had a strong effect on clinicians’ perceptions of patient care quality. In line with this, Mihail et al. [46], with a very similar theoretical focus, found that the relationship between HPWP and patient care quality was indirectly mediated by psychological empowerment. It was also demonstrated that HPWP has no statistically significant direct effect on the quality of care. The results of all these investigations [44,45,46] confirm that, without psychological empowerment, HPWP has a limited impact on patient care quality.

Furthermore, the investigation linking HPWP and empowerment with other organizational results does not stop. Abbasi et al. [47] conclude that psychological empowerment mediated the link between HPWP and knowledge sharing behavior. Likewise, in the study by Miyoung [48], psychological empowerment had a significant mediating effect on the relationship between HPWP and organizational citizenship behavior.

In this context, the relationships between HPWP and well-being have only begun to be considered a relatively short time ago [49]. The studies that include empowerment when it comes to explaining the effects of all these variables are still scarce. A rare exception is an article by Li and Lin [50]. Their results show that HPWP positively affects employees’ work well-being through psychological empowerment, but only when the leader’s trust in subordinates is high.

In the words of Spreitzer and Porath [51], managers need to pay attention to the physical and mental health of employees and create a happy workplace to promote the sustainable development of their organizations. We believe that, depending on the practices which the organization implants (structural empowerment) and how the employees internalize said practices (psychological empowerment), the levels of well-being that the employees experience could be very different. This is the principal question which we propose to investigate in this article.

## 2. Empowerment, Health, and Well-Being

Public health literature has proposed that one of the mechanisms explaining people’s health consists of their subjective experiences at work. There is evidence that employees’ multiple and varied experiences in the workplace impact their occupational health [52], and many of these experiences depend on the way they are managed and supervised [53]. In fact, some researchers in occupational health psychology argue that leadership should be considered an intervention area that can improve employee well-being (e.g., [54,55]).

In this context, an increasingly important aspect of leadership encompasses bosses’ empowering leadership behaviors towards their subordinates [56,57]. Some studies suggest that empowerment, in its different manifestations, can affect employees’ health and well-being [58,59,60,61]. However, Spreitzer [62] states that most research on the impact of empowerment has mainly been concerned with studying its effects on organizational performance and other individual outcomes, and that future research should explore a broader range of impacts, including health outcomes, because there is still little direct research linking empowerment and health. Laschinger and Read [63] reinforce this idea, arguing that, despite the research carried out to date, relatively few studies have examined the direct impact of empowerment on employees’ mental and physical health.

Therefore, we aim to investigate the relationships between structural and psychological empowerment, on the one hand, and their effects on psychological, physical, and social well-being, on the other, within organizations. The relationships between structural and psychological empowerment have previously been investigated, but not usually to analyze their effects on employees’ health and well-being. This study contributes to explaining how more general job characteristics, such as those defined by structural empowerment, can ultimately influence employee well-being. We argue that psychological empowerment is the variable that mediates in this relationship. Another contribution of our research is that it considers well-being at work from a broader perspective than most studies by including three dimensions of employee well-being, namely psychological, physical, and social according to Grant et al. [64], thus offering a more integrated view of these relationships.

Employee well-being is an important and current topic for organizations. However, due to its complexity, it is difficult to manage and measure. It can be defined as the overall quality of the way an employee experiences the job and functions at work [65,66]. The main dimensions of employee well-being, according to Grant et al. [64], include psychological well-being, which focuses on subjective experiences (e.g., satisfaction); physical well-being, which defines well-being in terms of bodily health (e.g., muscular pains, cardiovascular disease, blood pressure); and social well-being, which refers to relational experiences at work (e.g., support, reciprocity, cooperation). In the words of Grant et al. [64], this holistic definition is based on the healthcare, philosophy, psychology, and sociology literatures, and the level of agreement about these core elements of well-being (psychological, physical, and social) across the disciplines is surprising. Thus, we call attention to the multi-dimensional nature of employee well-being (empowerment can show different effects depending on the type of well-being considered), presenting a broader perspective of what is affected and how, and extending the range of important outcomes in the study of organizations.

Subordinates depend on the supervisor in a variety of ways that are essential in determining their levels of health and well-being [53,64,67]. Among other behaviors, bosses can delegate more power to their subordinates, increase their responsibilities, encourage independent decision-making, share information and knowledge with them, develop their skills, or encourage them to take risks or propose new ideas [68]. However, supervisors can also behave in the opposite manner, which would affect their employees’ health and well-being at work [69]. Therefore, it is not surprising that, after transformational leadership, empowerment leadership is the type of behavior that appeared more often in a scientific review carried out to determine the processes through which leadership behaviors affect employees’ psychological and physical well-being, thus becoming one of the most important mediating variables in this relationship [70].

Undoubtedly, the effects of empowerment, generally understood as the process of gaining control at work, have been studied the most in models of the effects of working conditions on health [71,72,73]. In the models by Siegrist [73] and Bakker and Demerouti [71], structural empowerment can be considered a set of organizational resources that employees need in order to meet the demands of their jobs [63]. A large body of research links these theoretical models to workers’ health and shows that the amount of control people have at work is related to their health [62]. To cite just a few classic examples, the results of a meta-analytic study show that perceived control over one’s work is associated with fewer physical and emotional health symptoms [74]. In addition, according to Johnson [75], the research shows that powerlessness in daily work life is strongly related to neurohormonal arousal, drug and alcohol use, mental distress, more chronic disease, and risk of early death, and it may even cause chronic illness.

However, the feeling of control at work is only one of the aspects of empowerment, which is a broader concept [76]. In relation to the study of empowerment in organizations, two complementary general approaches have mostly been used, structural empowerment and psychological empowerment [59,77,78,79]. The former conceives of empowerment as a set of structures, policies, and practices designed to decentralize power and authority throughout the organization (structural empowerment, hereinafter SE). The latter perspective, which is more psychological, focuses on the effects of these practices on employees’ initiative and motivation (psychological empowerment, hereinafter PE).

SE consists of a series of activities and practices implemented in the organization—and usually driven by management—that give subordinates power, control, and authority [80]. According to Laschinger et al. [81], this occurs when employees have access to lines of information, support, resources, and opportunities to learn and grow. SE has been found to predict job satisfaction and stress, among other variables [82,83]. Creating structurally empowering work environments is one way managers can reduce stressful working conditions and improve employees’ mental and physical health [63,77]. However, although some relationships can be established between SE and health and well-being, it is more difficult to explain how these effects occur, i.e., what mechanisms explain SE’s influence on health and well-being. We propose that one of the mechanisms mediating this relationship is PE.

PE exists when employees find their work to be meaningful and impactful and have enough autonomy and the necessary skills to be successful [76]. Several studies have addressed the relationships between SE and PE [83,84,85]. Currently, there is some agreement that both types of empowerment are necessary to successfully develop empowerment strategies within organizations. It is assumed that, on the one hand, organizations and their managers must provide employees with more power, share more information with them, and delegate more responsibilities (in the literature, this approach has been called a relational or mechanistic perspective and would coincide with SE). On the other hand, these strategies will mainly be successful when employees perceive that they are empowered (this approach has been called organic or psychological, and it is equivalent to the concept of PE).

The systematic review by Wagner et al. [86] showed that the studies reported a significant and positive relationship between SE (as the predictor variable) and PE (as the criterion variable), both at the general level and when the different factors that make up each of these constructs were analyzed separately (with some exceptions). Most of this work was carried out by Laschinger and her team, who, after completing more than 60 studies, highlighted the relevance of SE theory in the healthcare workplace. In sum, SE has been found to have a significant measurable impact when PE is also present [86]. Therefore, we propose the following hypothesis:

**Hypothesis** **1.** *Structural empowerment is positively related to psychological empowerment.*

As mentioned above, to date there is little direct research linking PE and health and well-being [62,63]. However, there are theoretical and empirical reasons to propose this relationship. Theoretically, it can be hypothesized that the four PE dimensions (meaning, competence, self-determination, and impact) have a negative relationship with poor health [87], and meaning has been proposed as a main protective factor against poor health. Moreover, as a protective factor, competence should result in a greater ability to deal with demands and, thus, buffer the effects of stress [88]. Likewise, some studies have argued that well-being is enhanced when individuals feel competent and autonomous [89]. Self-determination may be viewed as a form of autonomy, which is an important mechanism for reducing strain [90]. Impact can be considered the opposite of the theory of learned helplessness by Seligman, and so it may also be a buffer against poor health [60].

From an empirical perspective, the results obtained by Spreitzer et al. [60] in a two-sample study, one with supervisors and the other with entry-level employees, indicated that meaning and self-determination were significant predictors of job satisfaction, whereas meaning and competence were negatively related to work stress. Hochwälder and Brucefors [87] found that the meaning and competence dimensions were the ones most negatively related to poor health although, in general, the four empowerment dimensions were negatively related to the poor health indicators. Another study found that PE, especially the meaning, self-determination, and impact factors, had a small direct effect on mental and physical health, but an even greater indirect effect through enhanced job satisfaction [91].

In sum, as Orgambídez-Ramos et al. [92] state, jobs that have little meaning for employees seem to be associated with feelings of apathy and job dissatisfaction. Likewise, self-determination is a key element of intrinsic motivation that is closely related to autonomy, and so higher levels of both are associated with more positive assessments of work, thus increasing job satisfaction [59,60,81]. In conclusion, when workers perceive that they have autonomy and competence to perform tasks that are meaningful and have a high impact on the organization, they feel more satisfied with their jobs [59].

In addition, considering PE as one unique construct (without focusing on its different components), Holdsworth and Cartwright [91] propose that introducing PE in the workplace will produce a decrease in job stress and an increase in job satisfaction. In the study by Chung [93], PE had an inverse moderate correlation with job stress and a moderate correlation with job satisfaction. The results obtained by Tripathi and Bharadwaja [94] showed that PE was negatively related to perceived stress in a non-Western context (India) and using experimental methodology. In the meta-analytic review carried out by Seibert et al. [59], PE was positively associated with job satisfaction and negatively with employee strain, among other results. Finally, another investigated relationship is the one between empowerment, in its different expressions, and work engagement. As some studies point out, although the research is limited, PE has been found to be a significant predictor of work engagement [95,96,97]. Finally, Kim and Beehr [98] found especially strong relationships between PE and work engagement.

Taking all of this into account, we propose the following hypothesis linking PE to employees’ psychological well-being:

**Hypothesis** **2a.** *Psychological empowerment is positively related to job satisfaction and work engagement, and negatively related to stress.*

Regarding the relationships between PE and different aspects of well-being at work, very little research has analyzed the effects of empowerment in general on the physical health of employees [63]. Some studies, mentioned above, have found positive relationships between them [53,70,74,91,99]. Research by Kim and Beehr [98] also found that PE plays a role in individuals’ success in overcoming physical symptoms. However, appropriate indicators to measure an individual’s physical well-being have not always been used (see, for example, [53]). Therefore, it is necessary to further investigate this relationship, tentatively hypothesized as follows:

**Hypothesis** **2b.** *Psychological empowerment is positively related to physical well-being.*

Finally, people spend a lot of their time in contact with other people inside organizations. The quality of these relationships affects employees’ well-being (as, for example, in Karasek and Theorell’s Demands–Control–Support model [88]. We believe that the quality of these relationships can be influenced by the degree of empowerment employees experience in their organizations. Employees who feel competent when performing useful and important work that they can influence are more likely to maintain more positive relationships with each other than in the opposite case. Consequently, we propose that:

**Hypothesis** **2c.** *Psychological empowerment is positively related to social well-being.*

Many articles and reviews relate SE and PE to job satisfaction, especially in the hospital setting with nurses [58,100], although fewer studies have examined it in other areas. In the article by Laschinger et al. [101], SE had a direct and positive effect on PE, which in turn had a direct and positive effect on job satisfaction. Both SE and PE were significant predictors of job satisfaction. This study also found that PE had a direct and negative effect on job strain. In a later longitudinal study, Laschinger et al. [81] found that changes in SE had direct effects on changes in PE and job satisfaction. However, the changes in PE did not explain additional variance in job satisfaction beyond what was explained by SE. 

Both Spreitzer’s PE model [76] and Laschinger et al.’s SE model [101] propose the mediating role of PE between the more general characteristics of the organizational context, on the one hand, and work attitudes such as job satisfaction, on the other [92]. When SE is low, which translates into a lack of information at work or a lack of feedback, among other things, the experience of PE is reduced, which in turn is related to low scores on job satisfaction [58,92]. Furthermore, in their review, Wagner et al. [86] found that increased SE and PE are associated with greater job satisfaction. 

Studies have confirmed that PE mediates the effects of job characteristics (such as those stemming from SE) on work engagement [102,103]. Kimura [96] investigated the associations between SE, PE, and work engagement. As expected, higher levels of SE were associated with an increase in PE, which then led to higher work engagement. Likewise, PE has been found to be a mediator that explains the relationship between leadership and employees’ physical and mental health [53]. The results obtained by Butts et al. [104] indicate that PE fully mediates the relationship between high-involvement work practices and job satisfaction, and that job aspects reflecting high levels of empowerment are essential in alleviating job stress. Accordingly, we propose the following mediation hypothesis:

**Hypothesis** **3.** *Psychological empowerment mediates the relationship between structural empowerment and employee well-being.*

## 3. Method

### 3.1. Participants and Procedure

In this paper, we analyze the EU-27 data from the 6th European Working Conditions Survey (EWCS) conducted by the European Foundation for the Improvement of Living and Working Conditions in 2015 [105]. The aim of the EWCS is to provide an overview of the state of labor conditions in the European Union (EU) in order to identify major issues and changes affecting the workplace and contribute to better monitoring the quality of work and employment in Europe [16]. The EWCS questionnaire includes more than 100 items addressing a wide range of issues related to employment conditions and related variables. The questionnaire was created by Eurofound and tested in various ways to ensure that it provides a valid measurement of the concepts surveyed. The same questionnaire was used in all the countries involved, and data collection was uniform [105,106,107]. These data provide a unique and comparable source of information about working conditions in European countries. Data were collected in 2015 on a questionnaire administered face-to-face to a random sample of employees and self-employed people who were representative of the working population in the EU. In most countries, the target number of interviews was between 1000 and 2000 people. When a suitable sampling frame (register) with addresses/persons is not available for a country, the random route method is used to select the households and individuals. No quotas or other non-random solutions were implemented [107].

The sample consisted of 32,738 employees in EU-27 countries who, in the year 2015, were working as employees or employers/self-employed or relatives assisting on family farms or in businesses (52.4% male; 47.6% female). The missing data are not distributed completely at random (Little’s MCAR test: Chi-Square = 6527.915, DF = 2590, *p* = 0.000) in the variables selected for this research. We have chosen listwise deletion [108,109] because most of the variables present less than 2% missing data, with the exception of the following items: access to support (Q63e), access to information (Q71c), and the three items on the social well-being at work scale (Q70e, Q61a, and Q89d). The total sample size used in these analyses consists of 23,468 people we will consider together, in order to make statements at the European level.

### 3.2. Measures

To measure structural empowerment (SE), specified as a composite construct, we will follow the model proposed by Laschinger et al. [81,101], which is one of the most widely used models. Its original scale is composed of four central factors: opportunity, information, support, and resources. Access to opportunities refers to providing employees with opportunities for personal growth in organizations by increasing their knowledge and skills and encouraging their professional development on the job. Access to information measures the degree to which employees are informed about different aspects related to the organization’s policy, such as its current situation, values, or established objectives. Access to support measures the extent to which employees receive feedback and guidance, primarily from their supervisor, about how they are doing their jobs and how they could improve. Access to resources assesses the extent to which employees have the means and, above all, the time to do their jobs properly. Four items, one per factor, from the 6th EWCS [105] were used to measure these four factors. Support and resources were originally measured with an inverted 5-point Likert-type scale. Therefore, scoring was reversed, as Table 1 shows, so that a higher score always indicates higher SE.

To measure psychological empowerment (PE), specified as a composite construct, we will follow the Spreitzer model [76], undoubtedly the most widely used model in this field. It is also composed of four factors: meaning, competence, self-determination, and impact. Meaning implies congruence between an employee’s beliefs and values and the job requirements, and it is present when performing an important, valuable, or significant job. Competence refers to confidence in one’s job performance abilities. Self-determination refers to feelings of control over one’s work, which means enjoying certain autonomy, freedom, and independence to make decisions related to the assigned job. Impact refers to the sense of being able to influence important outcomes within the organization and doing useful work. Four items, one per factor, from the 6th EWCS [105] were used to measure these four factors. These items were originally rated on a 5-point Likert-type scale with values ranging from 1 = always to 5 = never. We recoded them, when necessary, as indicated in Table 1 to facilitate interpretation, such that a higher score indicates higher PE in all cases.

The dependent variables contemplate the three dimensions of employee well-being proposed by Grant et al. [64], introduced at the beginning of this article. Psychological well-being was measured based on three different constructs. The first one measures job satisfaction through a single item (as other researchers have done, for example, Lepold et al. [110]): “On the whole, are you very satisfied, satisfied, not very satisfied, or not at all satisfied with the working conditions in your main paid job?” (item Q88 from the 6th EWCS, 2016), which we recoded as 1 = Not at all satisfied to 4 = Very satisfied. Job stress was measured by another single item (see Houdmont et al. [111]): “You experience stress in your work” (item Q61m from the 6th EWCS), rated on a 5-point Likert scale ranging from 1 = always to 5 = never. To measure work engagement, we used a common factor scale with three items from the 6th EWCS [105]: “At my work, I feel full of energy” (Q90a), “I am enthusiastic about my job” (Q90b), and “Time flies when I am working” (Q90c). These last items were originally rated on a 5-point Likert scale with values ranging from 1 = always to 5 = never, which we reversed. In all cases, a higher score indicates greater psychological well-being at work: more job satisfaction, less stress, and greater work engagement.

Physical well-being was measured by asking about the existence of six symptoms of physical pain in the past 12 months, based on the 6th EWCS [105]: backache (Q78c), muscular pains in shoulder, neck and/or upper limbs (Q78d), muscular pains in lower limbs (Q78e), headaches, eyestrain (Q78f), injuries (Q78g), and overall fatigue (Q78i). Because they were all measured dichotomously, we operationalized this variable as the sum of these six symptoms and reversed the scores, so that a higher score indicates a higher level of physical well-being.

Finally, to measure *social well-being*, we also used a scale with three items from the 6th EWCS [105]: “Your colleagues help and support you” (Q61a), “There is good cooperation between you and your colleagues” (Q70e), and “I generally get on well with my work colleagues” (Q89d). All the items were originally measured with a 5-point Likert scale that we recoded so that a higher score always indicates a greater degree of social well-being.

As in Hochwälder and Brucefors [87], we consider sex, age, and occupation as control variables. These variables have been transformed into dummy variables to be introduced in the models, using as reference categories: male, 16–24 years old, and ISCO-0, respectively.

### 3.3. Analysis

Given that individual subjects respond to the items at a specific point in time using a relatively standardized set of question and response formats, the data are susceptible to Common Method Bias (CMB). Eurofound, in the design and data collection phases, took into account aspects that reduce the risk of CMB [112,113]: avoid ambiguous or complex items; use different choices of scale anchors; and avoid priming effects. Generally, no information is introduced about what the items are attempting to measure before the respondent views the items. In addition, Harman’s Single-Factor test specifies that all the items in the research model should be subjected to an exploratory factor analysis [113,114]. We loaded all the items into a principal components analysis with varimax rotation. Our results indicate the presence of five distinct factors with eigenvalues greater than 1.0, rather than a single factor. 

Because the model contemplates both composite constructs and common factors, we perform the analyses with the Partial Least Squares method [115,116,117,118], applying the mediation analyses [117,119,120] with the help of the SmartPLS version 3.3.2 [121]. 

## 4. Results

To check the validity of the measurement model, we use the recommended procedures for each type of construct [115,122,123]. Our explanatory variables have all been specified as composite constructs, and so we analyze the collinearity indicator, the significance, and the relevance of the indicator weights. Bootstrapping (5000 subsamples, no sign changes) was performed. All outer Variance Inflation Factor (VIF) values are below 1.57, which indicates that there are no collinearity problems. All weight values are significantly different from zero and can be considered relevant (the lowest was 0.182, see Appendix A).

The psychological and social well-being scales have been modeled as common factors. All item loadings are greater than 0.68. The composite reliability (Cronbach’s Alpha, rho A, and Composite Reliability) is greater than 0.7, and the variance extracted (AVE) is also above 0.5, indicating that they meet the commonly accepted cut-off values. Furthermore, there are no discriminant validity problems, analyzed with the Heterotrait–Monotrait Ratio (HTMT).

It should be noted that the use of goodness-of-fit indicators in PLS-SEM remains a controversial issue [124]. It is not clear that the NFI is an adequate indicator, and even among advocates of the use of SRMR, caution is also recommended regarding thresholds (0.08 or 0.10 depending on the author) since they are preliminary and further research is needed on the subject [125,126]. Taking these considerations into account, the fit values of our estimated model are SRMR = 0.051 and NFI = 0.859.

**Figure 1:**
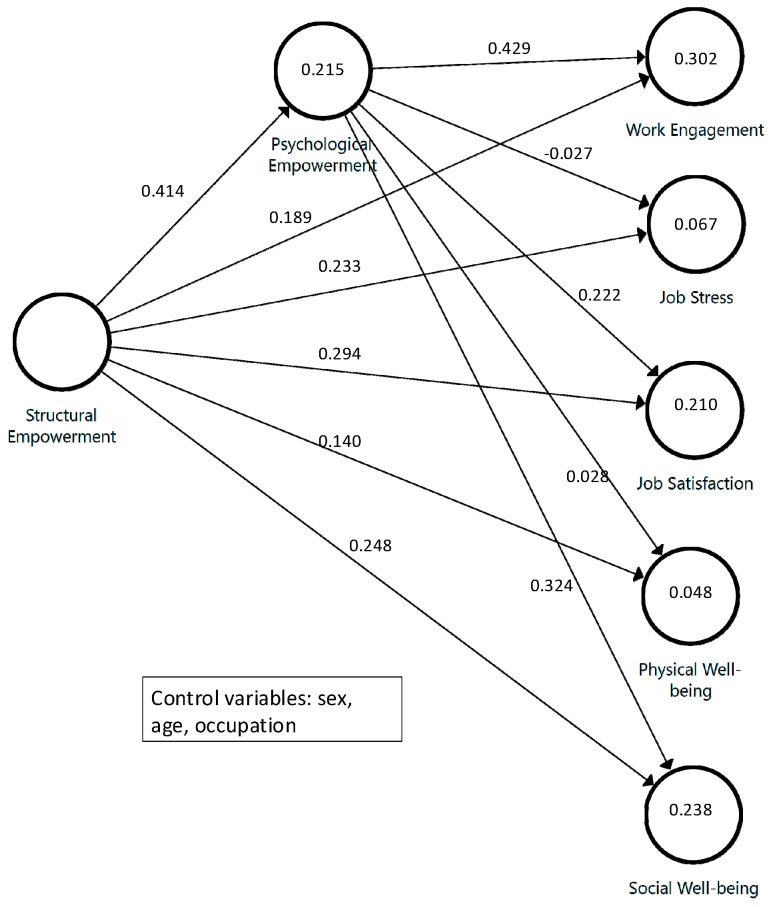
Simplified model representation. Values inside circles are R2. All paths are statistically significant. H3 was tested comparing direct paths from SE to well-being variables with the corresponding indirect paths (H1xH2a; H1xH2b and H1xH2c).

Appendix B presents the descriptive statistics for the items. The correlations between constructs (Table 2) are all positive, with values ranging from 0.047 to 0.519 (given the large sample size, all correlations are significantly different from zero). There are no collinearity issues in the structural model because all the VIF values are lower than 1.24, which is below the commonly accepted limit of 3.3. The analyzed model is shown in Figure 1 (see Appendix C for a view of the extended model).

The results (Table 3) support H1 because there is a significant path between SE and PE. Furthermore, PE has an R2 of 0.215, which can be considered a moderate-low value, but relevant. The paths between the two explanatory variables and all the dependent variables are also significant, but with different magnitudes. All SE paths range between 0.140 (the lowest, for the relationship with physical well-being) and 0.294 (the highest, for the relationship with job satisfaction). The paths from PE are also significant, but the paths linking this construct to physical well-being or job stress present values that are too low to be considered relevant; this can also be confirmed by the values from the bivariate correlation matrix (see Table 2). The other three paths have higher values and support H2a (except for stress) and H2c.

Given that there is a direct effect between SE and the employee well-being variables and that some of the direct PE relationships also seem relevant, we analyzed whether there is a mediation effect of PE (H3) or only a direct effect. To do so, we first check whether the indirect effect of SE is significant and relevant (see Table 3). All the indirect effects are significant, but given the large sample size, this is not surprising. However, two of the indirect effects (for physical well-being and job stress) are very small compared to the total effect. To test the strength of the mediation, we use the ratio of the indirect-to-total effect (VAF), obtaining values of 7.9% for physical well-being and −5% for job stress. Given that the values are low, if there is partial mediation for these variables, in the former it would be complementary, and in latter, it would be competitive because the multiplication of the direct and indirect paths has a negative sign [127]. The rest of the indirect effects, whose VAF values range between 24% and 49% of the total effect, can be considered a complementary partial mediation between SE and PE for the variables of job satisfaction, work engagement, and social well-being, providing support for H3 in relation to these variables. 

The control variables alone hardly explain the dependent variables. The paths are generally not relevant.

## 5. Discussion

New research is needed to better understand the management practices that impact employee health and well-being, both directly and indirectly [53]. Results from this study suggest that, in general, both SE and PE have direct relationships with employee psychological and social well-being variables. Furthermore, as we hypothesized, greater SE has a positive relationship with higher PE, which in turn translates into greater job satisfaction, work engagement, and social well-being (partial mediation). However, we did not find clear relationships between SE and PE and stress and physical well-being.

There is an ongoing debate about the effects of empowerment on the level of stress experienced by employees [59,62]. According to Tripathi and Bharadwaja [94], PE may cause high feelings of strain. When perceived autonomy is too high, employees might experience a lack of direction or excessive responsibility, causing them to experience stress. In addition, PE can increase stress if employees feel high frustration due to conflicting expectations from different parts of the organization. Moreover, employees who experience greater meaning at work are more likely to experience strain when things do not go as planned. Spreitzer et al. [60] stated that lower stress may lead to higher PE. Chung [93] also proposed that an optimal level of job stress can improve one’s PE. In their research, Orgambídez-Ramos et al. [92] aimed to analyze the impact of role stress on job satisfaction through PE (and not the opposite). Their hypothesis was that stress would negatively affect perceived PE levels, which in turn would reduce job satisfaction. Other authors have proposed theoretical models of the role of stress [53,101]. Likewise, stress has been shown to be associated with various physical and mental health disorders ranging from coronary heart disease to depression [128,129]. Numerous studies have concluded that job stress has a negative effect on job satisfaction and somatic symptoms such as headache and other physical problems, but some results have shown that dissatisfaction may be a source of stress [91]. The way these variables interact is still a controversial topic. We proposed the mediating role of PE in work stress, but it was not confirmed by our data, quite possibly for the reasons presented above. Only SE was found to have a direct effect on stress, but not high enough to provide a relevant explanation for what occurs with this variable.

No relevant relationships were found between the explanatory variables proposed in this study and physical well-being either. The relationships between PE and this variable, both direct and mediated, can be considered non-existent. Only a small direct effect of SE on physical well-being was detected. The explanation for these results could lie in the original proposal by Grant et al. [64]. These authors suggest that managerial practices (which involve favoring SE and PE) can often cause well-being trade-offs by enhancing one aspect of well-being while decreasing another aspect. For example, research indicates that work redesign practices can increase psychological well-being but decrease physical well-being. Other authors such as Khoreva and Wechtler [99] or Van De Voorde et al. [130] have also insisted on the possibility of trade-offs between the different dimensions of employee well-being when implementing different practices in organizations. Thus, these practices can be beneficial for variables related to psychological and social well-being, but they can simultaneously diminish employees’ physical well-being due to an increased workload and stress. As stated above, very few studies have analyzed the effects of empowerment on employees’ physical health [63]. Our results may help to explain why these relationships have not been addressed in the literature, or that they may not even really exist, or that it may not be feasible to study them with this type of cross-sectional data.

In our study, the hypotheses about the relationship between SE and PE (H1) and between PE and psychological (except for stress) and social well-being (H2a and H2c), as well as the relationship between SE and employee well-being mediated by PE (H3), were fulfilled, although the mediation was only partial.

Understanding the relationship between SE and PE will assist leaders in improving employees’ health and well-being at work [86]. This study reported a significant positive relationship between SE and PE, as other authors have also found [81,86,101]. Both are essential in sustaining empowerment in organizations, but each is incomplete by itself [84,85]. To improve workers’ health and well-being, it is necessary to introduce top-down changes in organizations (such as those fostered by SE), offering employees learning opportunities, sharing more information with them, and providing them with support and resources. At the same time, the desired effects will be greater if employees feel empowered (following a bottom-up perspective, that is, enhancing PE). 

SE, PE, and job satisfaction are closely linked, both directly and indirectly. Studies have revealed that increased PE can help leaders to increase their employees’ job satisfaction [59,60,101], although this result has not always been found [81]. We now have further confirmation that this relationship exists and is related to the idea that SE also has direct effects on job satisfaction and indirect effects through PE. Likewise, the direct relationship between PE and work engagement has been strengthened, as other studies have found [95,96,97,98], and we have also confirmed that PE strongly mediates the effect of SE on work engagement [96,103]. Similarly, and this is somewhat novel because previous studies have hardly addressed this issue, our results show a clear direct association between PE and social well-being, and they also show that PE strongly mediates the relationship between SE and social well-being (in addition to finding direct effects between them). Finally, PE has been found to function as a mediator and is partly responsible for the influence of SE on employees’ psychological well-being (except for stress). PE helps to explain the effects of the organization’s implementation of actions that favor SE on employees’ health and well-being.

In other words, managers can offer workers participation in workshops to improve product or service; this would allow workers to learn new things and access information. It would therefore elevate structural empowerment. However, this action would not have a clear repercussion on well-being if it is not accompanied by the presence of key managers in the final presentation of workshop results or a strengthening of the capacities of the operators to do their tasks. These actions would allow the worker to feel that their work is essential and that they can do their tasks well. That is to say, raise their psychological empowerment. These or other possible strategies could be put in place to improve the well-being of workers effectively.

## 6. Conclusions

In conclusion, increasing SE and PE has positive effects on workers’ health and well-being; specifically, it improves job satisfaction, work engagement, and social well-being. Although research on empowerment in organizations originally focused on its effects on increasing organizations’ effectiveness and quality, in recent years it has been strongly associated with organizational health [92]. Providing employees with information, support, resources, and opportunities to learn (SE) not only improves their performance but also has a positive impact on their health and well-being, and the variable that partially explains these results is PE. In fact, if we think about it in the opposite sense, how could a person not have health or well-being problems when doing a job that is not important or useful, that they cannot manage or change, and that they do not feel competent to perform well. There is increasing evidence that these relationships exist and have consequences.

### 6.1. Managerial Implications

Managers are often unaware of the consequences of their actions for the well-being of subordinates [64]. Managerial practices can cause synergies by affecting multiple dimensions of employee well-being. Our study points out that an adequate combination of SE and PE can produce positive relationships with employees’ job satisfaction, work engagement, and social well-being without producing trade-offs in terms of job stress and physical well-being (although the results indicate that there are no noteworthy improvements in these two variables, they do not worsen either). Wagner et al. [86] highlight the importance of designing specific workplace interventions based on empirical evidence showing that SE leads to PE, which culminates in measurable positive outcomes for workers’ health and well-being. Leaders must consider the relationship between SE and PE, both in general and when they are designing changes at all levels of the organization [102], because the accumulated evidence shows that it is possible to obtain positive long-term performance and well-being outcomes in the workplace.

In this regard, different practical applications of theoretical approaches to leadership (such as leader–member exchange, transformational leadership, etc.) have used PE as a mediating variable to explain the positive results obtained for employees’ health and well-being (e.g., [53,61]). Some authors have argued that high-involvement work practices positively influence empowerment because they can affect the cognitive states of PE [104,131], which in turn can improve employees’ levels of psychological and social well-being at work. However, this is not true in the case of their physical well-being [130] as the results of our study show. These studies reveal that both SE and practical applications of other leadership theories also draw on PE to explain the results obtained [70]. In other words, these interventions end up having a positive influence on employees’ health and well-being through PE. Some literature reviews even argue that the best strategy to improve occupational health is to empower employees through programs designed for this purpose [132]. Consequently, along with our results, almost all the previous scientific literature concludes that providing employees with information, support, resources, and opportunities to learn (the essence of the SE concept), encouraging them to value their work as meaningful and impactful, and showing them that they have adequate autonomy and the necessary skills to be successful (i.e., enhancing their PE) and form an effective integrated strategy to increase their levels of psychological and social well-being.

### 6.2. Limitations and Future Research

The results obtained in this study have to be interpreted with certain aspects in mind. First, the cross-sectional design does not allow causal relationships to be established between the variables, although this study suggests relationships of this type [92]. However, based on the theoretical models by Spreitzer [76] and Laschinger et al. [101], we can assume that these relationships exist between the study variables. Although it is possible that greater empowerment leads to better health, the relationship could be reversed, with healthier employees achieving greater levels of empowerment because they perform better. This issue cannot be clarified with this type of cross-sectional study; therefore, other studies based on experimental and longitudinal designs are needed [81].

The data were gathered from secondary data obtained with a questionnaire that was not specifically designed for this research. For this reason, the scales used to measure the constructs had to be adapted to the items available on the 6th EWCS [105]. Due to the close relationship between the objectives of the EWCS and those of this study, the items were appropriate. In addition, the sample was random, broad, and representative, which is not common in this type of study. This increases our confidence in the possibility of generalizing the results to all of Europe. Other limitations are that only self-reported answers by the respondents were used, and the data collection for all the variables was carried out in the same interview, which could lead to problems with common method variance. However, the use of the personal face-to-face interview for data collection and the statistical analyses performed seem to indicate that this bias is not likely. Moreover, taking advantage of the possibilities offered by the 6th EWCS [105] has allowed us to broaden the fragmented picture of employee well-being found in previous research, which only considered variables such as job satisfaction or stress in isolation, by grouping well-being into three dimensions (psychological, physical, and social) in order to offer a more integrated view of what well-being at work really means [64,99,130]. However, we have not selected all the variables that can be considered within each dimension of well-being, and so our results should be complemented by those from other current or future studies.

## Figures and Tables

**Table 1:** Measurement of the criterion variables.

Explanatory Variables (Composite Constructs)	Original Factors	Selected Item from the 6th EWCS [105]	Item Number from the 6th EWCS [105]	Codification (for This Article)
Structural Empowerment (SE)	Access to opportunity	Generally, does your main paid job involve learning new things?	Q53f	0 = No 1 = Yes
	Access to information	Does the following exist at your company or organization? A regular meeting in which employees can express their views about what is happening in the organization	Q71c	0 = No 1 = Yes
	Access to support	To what extent do you agree or disagree with the following statements? Your immediate boss provides useful feedback on your work	Q63e	Likert scale from 1 = Totally disagree to 5 = Totally agree
	Access to resources	You have enough time to get the job done?	Q61g	Likert scale from 1 = Never to 5 = Always
Psychological Empowerment (PE)	Meaning	I doubt the importance of my work (*reversed*)	Q90e	Likert scale from 1 = Always to 5 = Never
	Competence	In my opinion, I am good at my job	Q90f	Likert scale from 1 = Never to 5 = Always
	Self-determination	You can influence decisions that are important for your work	Q61n	Likert scale from 1 = Never to 5 = Always
	Impact	You have the feeling of doing useful work	Q61j	Likert scale from 1 = Never to 5 = Always

**Table 2:** Descriptive statistics and correlations between constructs.

	Mean	Min	Max	SD	Kurtosis	Skewness	(1)	(2)	(3)	(4)	(5)	(6)	(7)
(1) SE	2.230	0.490	2.962	0.463	0.253	−0.631	1.000						
(2) PE	4.053	1.000	5.000	0.616	0.552	−0.658	0.435	1.000					
(3) Work engagement	3.933	1.000	5.000	0.693	0.994	−0.737	0.381	0.519	1.000				
(4) Stress	3.085	1.000	5.000	1.114	−0.498	−0.093	0.197	0.047	0.151	1.000			
(5) Job satisfaction	3.067	1.000	4.000	0.683	0.742	−0.566	0.402	0.360	0.438	0.228	1.000		
(6) Physical well-being	3.852	0.00	6.000	1.736	−1.051	−0.304	0.160	0.090	0.203	0.234	0.247	1.000	
(7) Social well-being	4.339	1.000	5.000	0.621	2.187	−1.194	0.390	0.429	0.367	0.126	0.336	0.109	1.000

SE, Structural Empowerment; PE, Psychological Empowerment; SD, Standard Deviation.

**Table 3:** Summary of mediating effect tests.

Direct Effects	Path	Standard Deviation	*p*-Values	LCI 95%	UCI 95%	
SE -> PE	0.414	0.010	0.000	0.395	0.433	
SE -> Work engagement	0.189	0.012	0.000	0.166	0.212	
SE -> Stress	0.233	0.014	0.000	0.204	0.261	
SE -> Job satisfaction	0.294	0.012	0.000	0.270	0.318	
SE -> Physical well-being	0.140	0.012	0.000	0.117	0.165	
SE -> Social well-being	0.248	0.012	0.000	0.226	0.272	
PE -> Work engagement	0.429	0.011	0.000	0.406	0.451	
PE -> Stress	−0.027	0.013	0.041	−0.052	−0.002	
PE -> Job satisfaction	0.222	0.012	0.000	0.197	0.245	
PE -> Physical well-being	0.028	0.012	0.023	0.003	0.052	
PE -> Social well-being	0.324	0.012	0.000	0.300	0.348	
Indirect effects	Path	Standard Deviation	*p*-Values	LCI 95%	UCI 95%	VAF
SE -> Work engagement	0.178	0.006	0.000	0.165	0.191	48.50%
SE -> Stress	−0.011	0.005	0.041	−0.022	−0.001	−4.95%
SE -> Job satisfaction	0.092	0.005	0.000	0.081	0.103	23.83%
SE -> Physical well-being	0.012	0.005	0.023	0.001	0.022	7.89%
SE -> Social well-being	0.134	0.006	0.000	0.123	0.146	35.08%
Total Effects						
SE -> Work engagement	0.367	0.011	0.000	0.345	0.388	
SE -> Stress	0.222	0.013	0.000	0.196	0.248	
SE -> Job satisfaction	0.386	0.011	0.000	0.364	0.407	
SE -> Physical well-being	0.152	0.011	0.000	0.130	0.174	
SE -> Social well-being	0.382	0.011	0.000	0.361	0.404	
Model estimation	R2	R2Adjusted	BIC			
PE	0.215	0.215	−5621.046			
Work engagement	0.302	0.302	−8371.315			
Stress	0.067	0.067	−1567.765			
Job satisfaction	0.210	0.210	−5473.799			
Physical well-being	0.048	0.048	−1097.145			
Social well-being	0.238	0.238	−6325.081			

Bootstrapping based on *n* = 5000 sub-samples. LCI, Lower Confidence Interval; UCI, Upper Confidence Interval; BIC, Bayesian Information Criteria.

## Data Availability

https://www.eurofound.europa.eu/data/european-working-conditions-survey, accessed on 30 May 2021.

## Appendix A. Constructs Measurement Model

**Item Codes** **(see Appendix A)**	**Original Sample (Weights)**	**Standard Deviation**	***p*-Values**	**Weights LCI 95%**	**Weights UCI 95%**	**Original Sample (Loadings)**	**Standard Deviation**	***p*-Values**	**Loadings LCI 95%**	**Loadings UCI 95%**
Structural empowerment	Composite Construct									
Q63e -> SE	0.615	0.017	0.000	0.581	0.647	0.784	0.013	0.000	0.758	0.808
Q61g -> SE	0.506	0.019	0.000	0.467	0.542	0.616	0.019	0.000	0.578	0.651
Q71c -> SE	0.283	0.017	0.000	0.249	0.316	0.458	0.017	0.000	0.424	0.491
Q53f -> SE	0.234	0.019	0.000	0.197	0.272	0.330	0.021	0.000	0.289	0.371
Psychological empowerment	Composite Construct									
Q90f -> PE	0.273	0.019	0.000	0.237	0.310	0.520	0.019	0.000	0.482	0.557
Q61n -> PE	0.494	0.016	0.000	0.461	0.526	0.676	0.014	0.000	0.647	0.703
Q61j -> PE	0.537	0.019	0.000	0.498	0.574	0.810	0.012	0.000	0.786	0.832
Q90e -> PE	0.182	0.018	0.000	0.147	0.217	0.492	0.019	0.000	0.454	0.529
Work engagement	Common Factor. Cronbach’s Alpha = 0.716, rho A = 0.754, Composite Reliability = 0.839, Average Variance Extracted (AVE) = 0.636									
Q90a <- Work engagement	0.404	0.007	0.000	0.391	0.417	0.811	0.006	0.000	0.798	0.822
Q90b <- Work engagement	0.508	0.007	0.000	0.494	0.521	0.869	0.004	0.000	0.862	0.876
Q90c <- Work engagement	0.329	0.008	0.000	0.313	0.343	0.705	0.010	0.000	0.684	0.725
Stress	Single item Q61m									
Job satisfaction	Single item Q88R									
Physical well-being	Single item NoPhProblems6									
Social well-being at work	Common Factor. Cronbach’s Alpha = 0.697, rho A = 0.705, Composite Reliability = 0.833, Average Variance Extracted (AVE) = 0.625									
Q89d <- Social well-being	0.423	0.008	0.000	0.408	0.438	0.810	0.007	0.000	0.795	0.823
Q70e <- Social well-being	0.450	0.007	0.000	0.436	0.464	0.842	0.005	0.000	0.832	0.851
Q61a <- Social well-being	0.390	0.009	0.000	0.372	0.406	0.714	0.010	0.000	0.695	0.733
Bootstrapping based on *n* = 5000 sub-samples. LCI, Lower Confidence interval; UCI, Upper confidence interval.										

## Appendix B. Descriptive Statistics of the Items

**Construct**	**Code**	**Wording**	**N**	**Minimum**	**Maximum**	**Mean**	**SD**	**Skewness**	**Kurtosis**
SE	Q63e	Your immediate boss provides useful access to support on your work	26,825	1.00	5.00	3.80	1.114	−0.928	0.239
SE	Q61g	You have enough time to get the job done?	32,437	1.00	5.00	3.87	0.986	−0.879	0.476
SE	Q71c	Does the following exist at your company or organization: A regular meeting in which employees can express their views about what is happening in the organization	27,286	0	1	0.56	0.497	−0.237	−1.944
SE	Q53f	Generally, does your main paid job involve learning new things	32,480	0	1	0.73	0.446	−1.008	−0.983
PE	Q90f	In my opinion, I am good at my job	32,544	1.00	5.00	4.36	0.651	−0.957	1.911
PE	Q61n	You can influence decisions that are important for your work	32,088	1.00	5.00	3.30	1.308	−0.327	−0.954
PE	Q61j	You have the feeling of doing useful work	32,536	1.00	5.00	4.31	0.888	−1.466	2.240
PE	Q90e	I doubt the importance of my work (reversed)	32,532	1.00	5.00	4.11	1.065	−1.103	0.448
Psychological well-being:	Q90a	At my work I feel full of energy	32,678	1.00	5.00	3.81	0.807	−0.711	0.890
Work engagement									
Psychological well-being:	Q90b	I am enthusiastic about my job	32,655	1.00	5.00	3.86	0.929	−0.766	0.468
Work engagement									
Psychological well-being:	Q90c	Time flies when I am working	32,695	1.00	5.00	4.05	0.888	−0.846	0.589
Work engagement									
Psychological well-being: Stress	Q61m	You experience stress in your work	32,506	1.00	5.00	3.09	1.130	−0.046	−0.547
Psychological well-being:	Q88	On the whole, are you very satisfied, satisfied, not very satisfied, or not at all satisfied	32,601	1.00	4.00	3.09	0.680	−0.587	0.779
Job satisfaction		with working conditions in your main paid job?							
Physical well-being		Over the last 12 months, did you have any of the following health problems? (Sum of Q78 items below)	32,508	0	6	4.07	1.735	−0.490	−0.915
Physical well-being	Q78i_lt	Overall fatigue	32,628	0	1	0.65	0.476	−0.634	−1.598
Physical well-being	Q78g_lt	injury(ies)	32,653	0	1	0.92	0.266	−3.177	8.092
Physical well-being	Q78f_lt	headaches, eyestrain	32,657	0	1	0.64	0.479	−0.594	−1.648
Physical well-being	Q78e_lt	Muscular pains in lower limbs (hips, legs, knees, feet etc.)	32,662	0	1	0.70	0.455	−0.909	−1.173
Physical well-being	Q78d_lt	Muscular pains in shoulders, neck and/or upper limbs (arms, elbows, wrists, hands etc.)	32,676	0	1	0.58	0.492	−0.348	−1.879
Physical well-being	Q78c_lt	Backache	32,683	0	1	0.56	0.495	−0.262	−1.931
Social well-being at work	Q89d	I generally get on well with my work colleagues	29,772	1.00	5.00	4.39	0.735	−1.432	3.051
Social well-being at work	Q70e	There is good cooperation between you and your colleagues	26,897	1.00	5.00	4.30	0.771	−1.307	2.510
Social well-being at work	Q61a	Your colleagues help and support you	29,193	1.00	5.00	3.91	1.098	−1.000	0.452
Control variables	Sex	sex (men/women)				Frequency		Percent	
			1men			17,155		52.4	
			2women			15,576		47.6	
			Total			32,731		100.0	
			Missing			7		0.0	
Control variables	Age	age (16–24; 25–34; 35–54; 55 or more)				Frequency		Percent	
			16–24			2151		6.6	
			25–34			6702		20.5	
			35–54			17,809		54.4	
			55 or more			5926		18.1	
			Total			32,588		99.5	
			Missing			150		0.5	
Control variables	isco_08_1	ISCO_08 1-digit				Frequency		Percent	
			0Armed forces			101		0.3	
			1Managers			2467		7.5	
			2Professionals			5611		17.1	
			3Technicians			4842		14.8	
			4Clerical support			3727		11.4	
			5Service and sales			4982		15.2	
			6Skilled agricultural			813		2.5	
			7Craft workers			3982		12.2	
			8Plant operators			2693		8.2	
			9Elementary occupations			3212		9.8	
			Total			32,429		99.1	
			Missing			309		0.9	
		Valid N (listwise)	23,468						
SE, Structural Empowerment; PE, Psychological Empowerment; SD, Standard Deviation.									
